# Reducing the Consumption of Antibiotics: Would That Be Enough to Slow Down the Dissemination of Resistances in the Downstream Environment?

**DOI:** 10.3389/fmicb.2020.00033

**Published:** 2020-01-28

**Authors:** Christophe Merlin

**Affiliations:** Université de Lorraine, CNRS, LCPME, Nancy, France

**Keywords:** antibiotic resistance dissemination, antibiotic consumption, collateral antibiotic effect, resistance gene transfer, downstream environment

## Controlling the Consumption of Antibiotics

The emergence and dissemination of antibiotic resistance is now understood as an unavoidable aspect of bacterial evolution following the consumption of antibiotics (Courvalin, 2005). This dramatic phenomenon is well illustrated by the relationship existing between the occurrence of resistances and the consumption of antibiotics (Furuya and Lowy, 2006; Davies, 2007; Davies and Davies, 2010). Mechanistically speaking, the increasing occurrence of antibiotic resistant bacteria (ARB) has been widely attributed to the selection of resistant variants that pre-exist in susceptible communities (Andersson and Hughes, 2014). Such resistant bacteria are supposedly outcompeting the rest of the microbial communities in a context where antibiotics are administrated at relatively high levels, which means that local concentrations are well-over the Minimum Inhibitory Concentrations (MICs). Despite the fact that the increasing occurrence of antibiotic resistances among bacteria has been recognized decades ago as resulting from antimicrobial drug consumption, only recently has the seriousness of the situation been considered by international, national and local health organizations/agencies. This awareness led to series of reports and recommendations intending to educate and improve practices of health professionals and consumers, in order to preserve the effectiveness of our therapeutic potential (MSS, 2011; World Health Organization, 2015; O'Neil Report, 2016; EUR-Lex, 2018). Considering the correlation between antibiotic consumption and occurrence of resistances in bacteria (Davies, 2007), most recommendations proposed to take action in the public health and veterinary/farming domains by limiting the inappropriate exposure of bacteria to antibiotics in order to slow down a natural evolution toward resistance and its spread in the downstream environment in a One Health context (World Health Organization, 2015, 2017). Limiting the inappropriate exposure of bacteria to antibiotics implicitly means (i) reducing the need for antibiotics, which can be achieved with infection control measures that would limit the epidemic spread of resistant bacteria, and (ii) a better usage of antibiotics so as to reduce our overall antibiotic consumption when unnecessary.

Even if there is a great disparity between countries regarding the consumption of antibiotics (European Centre for Disease Prevention and Control (ECDC), 2017), change in practice remains difficult to implement when public health is concerned. In any case, taking action to reduce antibiotic resistance requires a coordinated and multi-sectorial approach combining political commitment, resources, specific governance mechanisms, and practical managements, as recently reported by World Health Organization (2018). In its 2018 reports, the ECDC indicated that the overall consumption of antibiotics in the EU did not significantly change in the community and the hospital sectors, while a few decreasing and increasing trends were observed for some countries over the 2013–2017 period. Changes in consumption were probably more visible in veterinary medicine. In a report covering the 2011–2016 period on veterinary antibiotics sale, the European Surveillance of Veterinary Antimicrobial Consumption related an overall decrease of 20% aggregated for 25 countries (European Surveillance of Veterinary Antimicrobial Consumption (ESVAC), 2018). This was tentatively explained by the implementation of policies and measures aiming at reducing the misuse of antibiotics. Even if the studied period is too short yet to draw robust conclusion, the first effects of such responsible-use campaigns start to be visible. In France for instance, an unprecedented national plan to reduce the antibiotic consumption in the animal sector has been initiated (Ecoantibio, 2017). This led to a drastic 39% reduction of antibiotic prescription in veterinary medicine in 6 years, all animals considered. The reduction was even stronger for critical antibiotics such as fluoroquinolones (81% reduction) and last generation cephalosporin (75% reduction). According to the French surveillance network of antimicrobial resistance in pathogenic bacteria of animal origin, these measures were followed by a net diminution of pathogenic ARBs (RESAPATH, 2016). As reported by the French National Public Health Agency (Santé Publique France, 2018), using data also presented by the European Food Safety Agency, the proportion of resistant *E. coli* for C3G went down from 16% to <2% in poultry between 2010 and 2017, which was dramatically increasing before 2010 (Bourély et al., 2018; European Food Safety Agency (EFSA), 2018; Santé Publique France, 2018). Although more results are necessary to comfort these results, they tend to demonstrate that a better use leading to a reduced consumption of antibiotics can rapidly result in a sensible decrease of the relative occurrence of ARBs. If several other reports are rather encouraging to pursue in that direction (Seppala et al., 1997; Aarestrup et al., 2001; Dutil et al., 2010), the relationship between occurrence of resistance and antibiotic consumption does not always follow this trend. Indeed, even if it is not the vast majority of the reported cases, stopping or increasing the consumption of a given antibiotic does not always result in the concomitant decrease or increase of the corresponding resistances, and this may vary according to the studied environment, the public/animal concerned, and the antibiotic and bacteria considered. For instance, Lai et al. (2011) reported a negative correlation between a decreasing consumption of cefotaxime and the rate of cefotaxime resistant-*Escherichia coli* pathogens isolated in a Taiwanese university hospital. Similar trends were reported for the consumption of ceftriaxone and ceftriaxone-resistant *E. coli* and *Klebsiella* spp. in a Turkish hospital setting (Altunsoy et al., 2011). Negative correlations between antibiotic consumptions and development of resistances can also work the other way around, and may depend on the bacterial species considered. In a Korean study covering six university hospitals, Kim et al. (2018) observed contrasted results following an increased consumption of fluoroquinolones, where the resistance rate for ciprofloxacin in *E. coli, Klebsiella pneumoniae*, and *Pseudomonas aeruginosa*, either increased, remained stable or decreased, respectively over an 8-years period. Surprisingly, the same authors also found negative correlation between decreasing consumptions of aminoglycosides and the resistance rate for third generation cephalosporins and ciprofloxacin, thus disconnecting a given drug consumption from its effect on the corresponding antibiotic resistance, at least for a few documented cases. To go further, it is worth noting that carbapenem-resistant *P. aeruginosa* could be isolated from animals that have not been previously treated with carbapenems. In this case, the resistance to carbapenem was attributed to an efflux pump dysregulation (rather than a carbapenemase) resulting from mutations possibly selected by disinfectants and other antibiotics in veterinary practices (Haenni et al., 2017), thus showing that resistant phenotypes can emerge independently from the presence of the corresponding antibiotics. On the other hand, the identification of antibiotic resistance genes in metagenomes from 30,000-years old sediments reminds us that resistance phenotypes and their corresponding genes probably existed before the so-called antibiotic era (D'Costa et al., 2011). Taken together, these observations clearly indicate that the emergence and the dissemination of antibiotic resistances in bacteria cannot solely be explained by a simple selective process occurring during antibiotics therapy, even if the latter is an important driving parameter in many instances.

## The Antibiotic Collateral Effects

Tackling the spread of antibiotic resistance will surely require a better usage of antibiotics in order to slow down the emergence of resistant variants associated to antibiotic therapies. But, considering the indispensability of antibiotics in modern medicine, antibiotic resistances will continue to be released in anthropogenically-impacted environments where ARBs can persist, accumulate, transfer their resistant genes (ARGs) to indigenous microbes, and finally re-enter the food chain and contaminate human and animal guts for a new round of selection (Davies and Davies, 2010). It should be noted here that the environment has been described as a reservoir of ARGs in several occasions (Berendonk et al., 2015). Considering that the dissemination of antibiotic resistances lies on the acquisition of resistance but also implies a transmission, and therefore a contact, between people, or with wastewater, or manure, or animals, tackling the dissemination of ARB and ARGs will surely require controlling both the usage of antibiotics but also the route of transmission, especially at the environmental level. With that respect, Collignon et al. (2018) recently proposed that the transmission of ARB and ARGs was probably the dominant contributor to consider for controlling antibiotic resistance, which implies to act at other levels than the antibiotic consumption as well.

The global reduction of consumption is not the sole important measure implemented by national and international organizations for better usage antibiotics. The classification of antimicrobial agents as critically important molecules for human health (WHO classification list), the restriction of their availability/delivery, and the confinement of particular antibiotic usages to human medicine are important measures aiming at preventing the emergence of particular resistances in the animal husbandry sector and their dissemination in the human health sector (EUR-Lex, 2018; OIE: World Organisation for Animal Health, 2018; World Health Organization, 2019). Nevertheless, confining the usage of a given antibiotic is likely to be of limited impact if collateral effects were to be observed between antibiotics of different nature on the emergence and the dissemination of unrelated ARGs. Lately, Scornec et al. (2017) demonstrated that *Tn916*, a mobile genetic element involved in the dissemination of an ARG for tetracycline, could exhibit a 1,000-fold increase of its transfer frequency when exposed to sub-inhibitory concentrations of tetracyclines, but also macrolides, lincosamides, and streptogramins. This means that not only sub-inhibitory concentrations of an antibiotic could stimulate the dissemination of its corresponding resistant gene, but that collateral stimulation by other antibiotics is also possible. This tends to rule out the effectiveness, at least partially, of any measure that would be based on confining the usage of cross-reacting antibiotics. On another ground, the use of trace metal elements such as zinc oxide or copper sulfate, is frequently used as an antibiotic alternative to promote growth of livestock and poultry. Consequently to such practices, several authors reported a concomitant increase of ARB and ARGs that are likely to result from co-selective processes, as ARGs and metal resistance genes can collocate on the same genetic entities (mobile genetic elements) (Hasman et al., 2006; Yin et al., 2017; van Alen et al., 2018).

## Conclusion

Collateral effects of antibiotics at sub-inhibitory concentrations and trace metal elements clearly highlight the fact that the antibiotic resistance risk should not be associated with the sole antibiotic therapy practices, and should rather be considered as a multifactorial problem where co-selection and stimulation of horizontal gene transfer also fully applies. Further in depth epidemiological studies should allow determining the extent of such collateral effects outside the context of a Petri dish, and may explain why some antibiotic resistances escape any reduction of occurrence while reducing the corresponding antibiotic consumption. On the other hand determining exhaustively which antibiotic molecules exhibit collateral effects, and at which concentrations, would be an additional step toward antibiotic risk assessment, whether for therapeutic practices or for the effect of antibiotics once diluted in the downstream environments.

## Author Contributions

CM wrote this opinion paper.

### Conflict of Interest

The author declares that the research was conducted in the absence of any commercial or financial relationships that could be construed as a potential conflict of interest.
